# Haematological Indices and Antioxidant Enzyme Activity in Ghanaian Stroke Patients

**DOI:** 10.1155/2022/1203120

**Published:** 2022-03-03

**Authors:** Richard Harry Asmah, Pomah Sackey, Patrick Adjei, Timothy N. Archampong, Seth Attoh, Derek Doku, Marjorie Quarchie, Felix Botchway, David Adedia, Eric Sampene Donkor

**Affiliations:** ^1^Department of Biomedical Sciences, School of Basic and Biomedical Sciences, University of Health and Allied Sciences, Ho, Ghana; ^2^Department of Medical Laboratory Sciences, School of Biomedical and Allied Health Sciences, University of Ghana, Ghana; ^3^Department of Medicine and Therapeutics, University of Ghana Medical School, Ghana; ^4^Division of Pathology, 37 Military Hospital, Accra, Ghana; ^5^Child Health Department, Korle-Bu Teaching Hospital, Accra, Ghana; ^6^Department of Basic Sciences, School of Basic and Biomedical Sciences, University of Health and Allied Sciences, Ho, Ghana; ^7^Department of Medical Microbiology, University of Ghana Medical School, Ghana

## Abstract

**Background:**

Stroke is a cardiovascular disorder causing mortality globally and long-lasting harm worldwide. The disease occurs when the blood flow to the brain is either interrupted or blocked. This disruption leads to the increase in reactive oxygen species (ROS), especially superoxide free radicals, resulting in oxidative stress. The superoxide radicals are removed by superoxide dismutase (SOD), a key antioxidant enzyme. In this work, we investigated haematological indices and superoxide dismutase enzyme activity in Ghanaian patients with stroke and healthy control participants.

**Materials and Methods:**

Thirty stroke patients attending a stroke clinic and thirty apparently healthy control participants were recruited into the study. Blood samples were collected to determine haematological indices and SOD enzyme activity in red blood cells.

**Results:**

The stroke patients had significantly high blood parameters such as white blood cell (*p* < 0.001), neutrophil (*p* < 0.001), lymphocyte (*p* = 0.003), and eosinophil (*p* < 0.001) comparing with study participants without stroke, who were the control group in the study. Other blood parameters such as red blood cell, (*p* < 0.001), haemoglobin (*p* < 0.001), and haematocrit (*p* < 0.001) levels and mean cell haemoglobin concentration (*p* = 0.030), platelet (*p* = 0.010), and plateletcrit (*p* = 0.027) were high in stroke patients comparing with study control participants and statistically significant. Blood lymphocyte levels observed in stroke patients correlated negatively and significantly with SOD activity levels. SOD activity levels were significantly lower in stroke patients compared with the control group (*p* < 0.001). Low values of the antioxidant enzyme SOD activity levels, lymphocytes, and high values of plateletcrit were significant predictors of stroke.

**Conclusion:**

Haematological parameters such as WBC, lymphocyte, platelet levels, and red cell indices were significantly different in the stroke patients being studied. There was negative correlation between lymphocyte significantly with SOD activity and high oxidative stress in stroke patients compared with the control group. Lymphocytes and plateletcrit levels were also good predictors of the occurrence of stroke.

## 1. Introduction

Stroke is a cardiovascular disorder causing mortality globally and long-lasting harm worldwide [1, 2]. The disease is a leading cause of adult disability [3], the second cause of dementia [4], and the third cause of mortality in most developed countries such as sub-Saharan Africa [5]. These conditions result in about 70 percent of death and 87% of disabilities in low- and middle-level-income countries [1]. According to the World Health Organization, stroke is the second leading cause of death among people over 60 years of age and the fifth leading cause among those 15 to 59 years of age [6]. In Africa, especially sub-Saharan Africa, currently, there is an increase in stroke cases in middle-age lives [7, 8]. The reported annual incidence of stroke in Africa was reported as 316 per 100,000 individuals [1]. Stroke is classified mainly into two types: ischaemic and haemorrhagic [9]. There are reports of high-level small-vessel disease- (ischaemic-) related stroke and intracerebral lesion on the continent of Africa than elsewhere in the world [10]. Hypertension remains the most important risk factor in Africa including other such as diabetes, stress, dyslipidaemia, cardiac disease, and obesity [11]. There are gaps in the general understanding of stroke care, practice, and policy in Africa [1]. Research insights into the disease would offer useful comprehension into stroke prevention and care.

Reactive oxygen species produced during ischaemic and perfusion phases in acute ischaemic stroke can lead to brain injury [12]. Also, unstable haemostasis in the brain causes the increased production of free radicals, especially reactive oxygen species such as superoxide. Increasing evidence suggests that reactive oxygen molecules are closely linked to tissue injury during stroke [13]. Elevation in reactive oxygen species can be injurious and also lead to adverse changes to biomolecules such as lipids, proteins, DNA, RNA, and enzymes in cells [14]. These changes include cell protein denaturation, DNA breaks, and alterations of cell membrane fluidity and eventually may lead to cell death. Furthermore, an increase in reactive oxygen species also leads to adverse oxidative stress situations in the stroke patients [15]. Biological functions such as apoptosis, necrosis, and phagocytosis are mediated by reactive oxygen species. These reactive metabolites are selectively neutralized by the body's defensive mechanism. Principal defensive agents are antioxidant enzymes such as superoxide dismutase, catalase, and glutathione peroxidase [16].

The imbalance caused by oxidative stress results in red blood cell dysfunction, platelet destruction, and tissue injury. In stroke patients, such abnormalities may affect blood cell function as well as the coagulation system, leading to anaemia, infection, and hypercoagulability [17]. In the pathogenesis of acute ischaemic stroke, low-molecular weight antioxidants are involved in the reactions with free radicals generated during physiological cellular reactions resulting in oxidative stress [18]. The antioxidant enzyme superoxide dismutase and its derivatives cause vasodilation by opening potassium channels as well as deterioration of vascular reactivity [19]. Cerebrovascular thrombosis and subsequent cerebral perfusion injury in stroke necessitates constant monitoring of haemostatic parameters in stroke patients for effective management and care. In this study, we investigated haematological indices and superoxide dismutase activity in Ghanaian patients with stroke and study participants without stroke.

## 2. Materials and Methods

### 2.1. Study Design and Site

This cross-sectional study was conducted between May and July 2017 recruiting 60 study participants following informed consent by random sampling. Of these, thirty were diagnosed with stroke and were on admission at the stroke clinic of Korle-Bu Teaching Hospital (KBTH). The control group (*n* = 30) comprised of persons without clinical evidence of stroke or any history of the disease which were recruited from relatives of patients in the out-patient clinic of the healthcare facility. Structured questionnaire was used to collect data about subject demographic, life style, alcohol intake, disease condition, treatment history, and family history of stroke.

### 2.2. Inclusion Criteria

Inclusion criteria: patients (males and females) between the ages of 20 and 80 who were clinically diagnosed with stroke and who are in recovery during the first three to four months, were included in the study. Apparently, healthy subjects between the ages of 20 to 80, without any clinical evidence of stroke, served as control participants.

### 2.3. Clinical Evaluation

The clinical history of all participants including demographic data, diagnosis, treatment history, and family history of stroke was recorded.

### 2.4. Blood Sample Collection

Three millilitres of venous blood was collected from each study participant into an ethylenediaminetetraacetate (EDTA) tube for full blood count analysis. The blood samples were then centrifuged at 4000 rpm for 10 minutes at 4°C. Red blood cells obtained were stored by refrigeration for further analysis, while plasma and buffy coat were aliquoted into separate microcentrifuge tubes and stored at −20°C until use.

### 2.5. Sample Analyses

Full blood count was carried out at the central laboratory (KBTH), and SOD activity assay was carried out at the laboratories of the School of Biomedical and Allied Health Sciences, University of Ghana, Accra, Ghana.

### 2.6. Full Blood Count

The haematological parameters including white blood cell (WBC), neutrophil (NEUT), lymphocyte (LYM), eosinophil (EOS), basophil (BAS), red blood cell (RBC), count, haemoglobin concentration (HGB), haematocrit (HCT), mean cell volume (MCV), mean cell haemoglobin (MCH), mean cell haemoglobin concentration (MCHC), and platelet concentration (PLT) were determined by an automated blood analyzer (Mindray BC-5300, China).

### 2.7. Superoxide Dismutase (SOD) Assay

Superoxide dismutase activity was determined using a commercial kit, SOD Assay Kit-WST (Sigma-Aldrich, St. Louis, MO, USA), following the manufacturer's protocol. Red blood cell lysate of the study participants was used. Prepared briefly as follows, 50 *μ*l of RBC pellets from each participant was lysed on ice for 5 min using double-distilled ice-cold sterile water followed by centrifugation at 13000 g for 15 min at 4°C. The supernatant was diluted 100x using double-distilled ice-cold sterile water. The manufacturer's protocol required that twenty microlitres of the supernatant from each sample processed is added to the wells of a 96-microtitre plate. Three wells on the microtitre plate are designated as blanks 1, 2, and 3. Two hundred microlitres of water-soluble formazan (WST) working solution was added to each well and mixed well. Also added was 20 *μ*l of sample dilution buffer to blanks wells, labeled 2 and 3. Twenty microlitres of enzyme working solution was added to samples and blank 1 except blanks 2 and 3. The microtitre plate was mixed thoroughly by incubating briefly on a rocking platform. The reaction mixture was incubated at 37°C for 20 min and absorbance was read at 450 nm using a spectrophotometer (LabSystems Multiskan MS, LabSystems, Finland). SOD activity was calculated per formula described by the kit manufacturer. The SOD activity assays were done in duplicates [20, 21].

### 2.8. Statistical Analysis

The data obtained from the study was entered into Microsoft Excel 2010 and analyzed statistically using XLSTAT, R and Statistical Package for Social Sciences (SPSS) 25.0 software for Windows to arrive at the summary statistics of mean and standard deviation for numerical variables and proportions for categorical variables. An independent *t*-test was used to determine the mean differences between quantitative variables. Pearson's correlation analysis was used to look at association between variables. A *p* value less than 0.05 was interpreted as significant. Receiver operating characteristic (ROC) analysis was used to assess ability of SOD enzyme activity and various haematological indices (lymphocyte (LYM), mean cell haemoglobin, (MCH), mean cell haemoglobin concentration (MCHC), monocyte (MON), mean platelet volume (MPV), platelet distribution width (PDW), platelet (PLT), total white blood cell (WBC), neutrophil (NEUT), eosinophil (EOS), basophil (BAS), red blood cells (RBC), haemoglobin (HGB), haematocrit (HCT), mean cell volume (MCV), and plateletcrit (PCT)) in predicting stroke. The ROC curve, area under the curve, sensitivity, specificity, and accuracy of prediction were the criteria used. Sensitivity measures the probability that a person has stroke given that the person actually has stroke, whilst specificity measures the probability that a person does not have stroke given that the person does not have stroke [22].

### 2.9. Ethics

Ethical clearance was sought from the ethics and protocol review committee of the School of Biomedical and Allied Health Sciences (SBAHS), University of Ghana, prior to the commencement of the study and enrollment of subjects. Also, permission was sought from the head of the stroke unit of Korle-Bu Teaching Hospital before the start of the study. Written informed consent was obtained from the study participants.

## 3. Results

### 3.1. Demographic and Clinical Characteristics

Tables 1 summarizes the distribution of demographic and clinical characteristics of study participants. The numbers of males in the study were 20 (66.7%) and 18 (60%) for stroke patients and controls, respectively (Table 1). The numbers of females in the study were 10 (33.3%) and 12 (40.0%) for stroke patients and controls, respectively (Table 1). The mean age of the stroke group was significantly higher than that of the control group (*p* = 0.003). The mean period of stroke for the cases was 1.93 ± 1.0 weeks (Table 1). Risk factors identified were hypertension 13 (43.3%), diabetes mellitus 10 (33.3%), physical inactivity 3 (10%), and smoking 2 (6.7%). The spectra of stroke cases were haemorrhagic 10 (33.3%), 10 (33.3%), recurrent 2 (6.7%), infarctive 6 (20%), and transient ischaemic attack 2 (6.7%).

### 3.2. White, Red Blood Cell and Platelet Parameters

In Table 2, stroke participants had significantly higher numbers of white blood cell (WBC) (*p* < 0.001), neutrophil (NEU) (*p* < 0.001), lymphocyte (LYM) (*p* = 0.003), and eosinophil (EOS) (*p* < 0.001) as compared with the control group. However, for mean monocyte (MON) (*p* = 0.4570), as well as basophil (BAS) (*p* = 0.226), the difference between stroke and control groups was not significant. Table 2 also shows the mean platelet volume (MPV) (*p* = 0.523) and mean platelet distribution width (PDW) (*p* = 0.235). Differences between stroke patients and control group were not statistically significant. For the mean platelet count (PLT) (*p* = 0.010) and mean plateletcrit (PCT) (*p* = 0.027), differences between stroke patients and controls were statistically significant. In Table 3, red blood cell (RBC), (*p* < 0.001) haemoglobin (HGB) (*p* < 0.001), and haematocrit (HCT) (*p* < 0.001) levels and mean cell haemoglobin concentration (MCHC) (*p* = 0.030) were statistically significant comparing stroke patients with the control group. Mean cell volume (MCV) (*p* = 0.583) and mean cell haemoglobin (MCH) (*p* = 0.207) levels were not significantly different between stroke patients and the control group.

### 3.3. Superoxide Dismutase Activity

**Figure 1:**
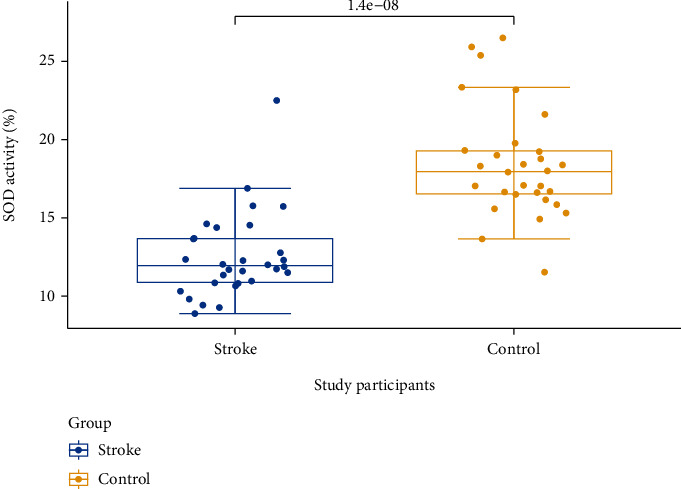
Box plot distribution of SOD activity among control and stroke study participants.

Figure 1 shows a box plot with mean SOD activity after analysis of blood samples. Superoxide dismutase activity among the control group (*n* = 30; 18.46 ± 3.53) was significantly (*p* < 0.001) compared with that among stroke patients (*n* = 30; 12.54 ± 2.74).

### 3.4. Correlation between SOD Activity and Blood Parameters

As seen in Table 4, neutrophil (NEU) (*r* = −0.135), lymphocyte (LYM) (*r* = −0.326), and monocyte (MON) (*r* = −0.015) correlated negatively with SOD activity. WBC (*r* = 0.018), EOS (*r* = 0.005), and BAS (*r* = 0.137) correlated positively with SOD activity. The correlation between SOD activity and lymphocyte was significant (*p* = 0.014). In Table 4, the SOD activity correlated negatively with MPV (*r* = −0.011) and PCT (*r* = −0.041) and there was positive correlation between SOD activity with PLT (*r* = 0.044) and PDW (*r* = 0.070). In Table 4, HGB (*r* = 0.137), HCT (*r* = 0.137), MCV (*r* = 0.321), MCH (*r* = 0.330), and MCHC (*r* = 0.054) positively correlated with SOD activity. There were significant correlations between SOD activity levels with MCV (*p* = 0.015) and MCH (*p* = 0.013) in stroke patients.

### 3.5. Receiver Operator Characteristic Curve Analysis

**Figure 2:**
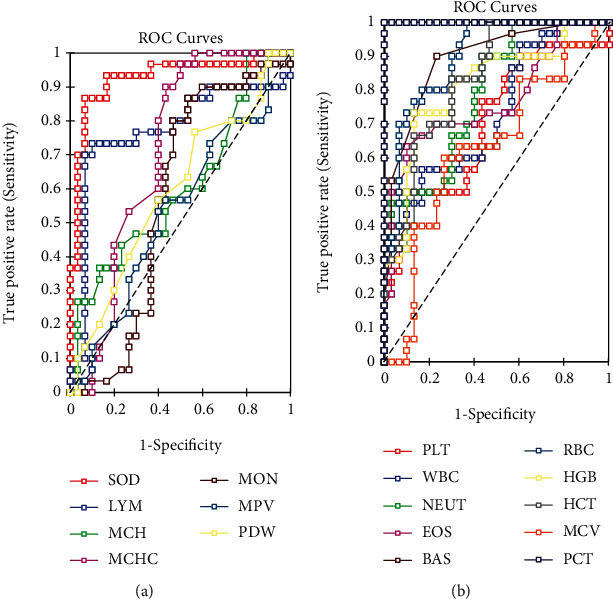
Receiver operator characteristic curve for predicting stroke.

Lower values of superoxide dismutase (SOD) enzyme activity, lymphocyte (LYM), mean cell haemoglobin (MCH), mean cell haemoglobin concentration (MCHC), monocyte (MON), mean platelet volume (MPV), and platelet distribution width (PDW) were related with stroke, whilst SOD enzyme activity, LYM, and MCHC were significant predictors of stroke. SOD enzyme activity (0.9000) had the highest accuracy in predicting stroke and was followed by LYM (0.8167). They also had higher area under curve (AUC) values as well as sensitivity and specificity (Table 5; Figure 2(a)). Higher values of platelet (PLT), white blood cell (WBC), neutrophil (NEUT), eosinophil (EOS), basophil (BAS), red blood cells (RBC), haemoglobin (HGB), haematocrit, (HCT), mean cell volume (MCV), and plateletcrit (PCT) were related with stroke. MCV was not a significant predictor of stroke. Other markers were significant determinants of stroke, with PCT being the highest predictor of stroke having the highest sensitivity, specificity, accuracy, and AUC values (Table 5; Figure 2(b)).

## 4. Discussion

The economic impact of stroke causes financial cost to a country's health care system as well as loss of income and productivity on the part of those affected, either directly by the disease or indirectly as caregivers to those with stroke [23]. In this work, we investigated haematological indices and superoxide dismutase enzyme activity in Ghanaian patients with stroke and healthy control participants. White blood cells in the stroke patients and control participants were significantly different as seen in Table 2. Hu et al. [24] reported that high white cell indices were predictive of stroke severity and the results of the study was in agreement with the present one. In addition, the stroke patients with haemorrhagic stroke had elevated WBC. There are reports in a study that elevated levels of WBC and lymphocytes could be due to atherosclerosis, an inflammatory process, and could result in cerebral infarction [25]. There were significant differences in blood parameters such as mean cell haemoglobin concentration (MCHC) and haematocrit (HCT) levels (Table 3). There are reports that an increase in RBC above normal levels thickens blood and makes clots likely and increases the risk for stroke as this contributes to cerebrovascular blood viscosity [26]. This could possibly explain the high levels of RBC indices observed in the stroke patients in this study; increased levels of RBC indices are reported as risk factors of ischaemic stroke, cardiovascular disease, and venous thromboembolism [27]. There was significant difference in the platelet number, with stroke patients having high values (Table 2). Agreeing with reports that increase in platelet levels may imply augmented platelet aggregation and activity in early stroke, and the value of platelet counts may possibly be predictive of an event of stroke [28].

Reduced SOD activity levels in this study observed in the stroke patients (Figure 1) agree with those reported by Cherubini et al. [29]. Additionally, there was also negative correlation between SOD activity levels and lymphocyte levels that was significant (Table 4). SOD activity levels weakly correlated with neutrophil, eosinophil, and monocyte levels. This could be explained by reports that leucocytes are endogenous sources of ROS and could possible explain the low SOD activity levels in the stroke patients [12]. The sensitivity and specificity of biological markers are useful in predicting stroke if their sum is greater or equal to 1.5 using receiver operator characteristic analysis [30]. We also assessed the stroke predictive ability of haematological indices and superoxide dismutase activity using the sensitivity, specificity, accuracy, and area under the curve (Table 5, Figures 2(a) and 2(b)). For biological markers where smaller test values indicate more positive test, the area under curve (AUC) values show that SOD enzyme activity was a better predictor of stroke. Lymphocytes were also a good predictor of stroke, looking at sensitivity, specificity, and AUC values. For markers where larger test values indicate a more positive test, the AUC values showed that PCT excellently predicts stroke, with very high sensitivity and specificity values. However, the study has limitations including the small sample size used and we could not analyze other antioxidant enzymes such as catalase and glutathione peroxidase.

## 5. Conclusion

Haematological parameters such as WBC, lymphocyte levels, and red cell indices were significantly different in the stroke patients being studied. There was negative correlation of lymphocytes significantly with SOD activity and high oxidative stress in stroke patients compared with the control group. Lymphocytes and plateletcrit levels were also good predictors of the occurrence of stroke. Further investigations of these parameters would allow better clinical management and care of stroke outcome.

## Figures and Tables

**Table 1:** Demographic parameters of patients.

Variable	Stroke patients (*n* = 30)	Controls (*n* = 30)	*p* value
Mean age (SD)	57.0 (16.2)	41.6 (14.4)	0.003^∗^
Duration of stroke (weeks) mean (SD)	1.93 (1.0)		

Age groups	Stroke patients (%)	Controls (%)	
20–29	0 (0.0)	7 (23.3)	
30–39	7 (23.3)	10 (33.3)	
40–49	4 (13.3)	6 (20.0)	
50–59	1 (3.4)	2 (6.7)	
60–69	11 (36.7)	3 (10)	
70–79	7 (23.3)	2 (6.7)	

Gender (%)			
Male	20 (66.7)	18 (60.0)	
Female	10 (33.3)	12 (40.0)	

SD: standard deviation; %: percentage. Values are presented as mean (standard deviation) or value (percentage or range). *p* value was set at 0.05.

**Table 2:** White blood cell and platelet parameters.

Variable	Mean (SD)		*p* value
(×10^9/L)	Stroke patients (*n* = 30)	Control (*n* = 30)	
WBC	8.72 (4.61)	5.05 (2.93)	<0.001^∗^
NEU	7.6 (6.7)	2.57 (2.4)	<0.001^∗^
LYM	1.5 (0.85)	2.16 (0.66)	0.003∗
MON	1.04 (4.34)	0.44 (0.64)	0.457
EOS	0.27 (0.19)	0.12 (0.09)	<0.001^∗^
BAS	0.14 (0.54)	0.02 (0.01)	0.226
PLT	267.58 (103.0)	202.97 (77.6)	0.008^∗^
MPV (fL)	9.76 (1.04)	9.96 (1.35)	0.523
PDW	16.15 (0.36)	16.31 (0.60)	0.235
PCT (mL/L)	2.74 (.63)	0.21 (0.09)	<0.001^∗^

WBC: total white blood cell; NEU: neutrophil; LYM: lymphocyte; MON: monocyte; EOS: eosinophil; BAS: basophil; PLT: platelet; MPV: mean platelet volume; PDW: platelet distribution width; PCT: plateletcrit; SD: standard deviation. Values are presented as mean (standard deviation). ^∗^Mean difference is statistically significant at *p* < 0.05.

**Table 3:** Red blood cell parameters.

Variable	Mean (SD)		*p* value
	Stroke patients (*n* = 30)	Control (*n* = 30)	
RBC (×10^12/L)	5.57 (1.79)	4.18 (1.02)	<0.001^∗^
HGB (g/dL)	14.5 (1.9)	11.4 (3.4)	<0.001^∗^
HCT	0.48 (0.06)	0.37 (0.1)	<0.001^∗^
MCV (fL)	91 (6.3)	89.73 (11.0)	0.583
MCH (pg)	27.6 (1.93)	28.3 (2.3)	0.207
MCHC (g/dL)	30.37 (1.0)	31.59 (2.76)	0.030^∗^

*n*: sample size; RBC: red blood cells; HGB: haemoglobin; HCT: haematocrit; MCV: mean cell volume; MCH: mean cell haemoglobin; MCHC: mean cell haemoglobin concentration; SD: standard deviation. Values are presented as mean (standard deviation). ^∗^Mean difference is statistically significant at *p* < 0.05.

**Table 4:** Correlation between SOD activity, WBC, platelets, and RBC parameters.

Correlation test	Pearson's *r* value	*p* value
*SOD* ^∗^ *WBC*	0.018	0.891
*SOD* ^∗^ *NEUT*	−0.135	0.310
*SOD* ^∗^ *LYM*	−0.326^∗^	0.014^∗^
*SOD* ^∗^ *MON*	−0.015	0.910
*SOD* ^∗^ *EOS*	0.005	0.970
*SOD* ^∗^ *BAS*	0.137	0.301
*SOD* ^∗^ *PLT*	0.044	0.740
*SOD* ^∗^ *MPV*	−0.011	0.934
*SOD* ^∗^ *PDW*	0.070	0.597
*SOD* ^∗^ *PCT*	−0.041	0.757
*SOD* ^∗^ *RBC*	−0.064	0.629
*SOD* ^∗^ *HGB*	0.137	0.300
*SOD* ^∗^ *HCT*	0.137	0.301
*SOD* ^∗^ *MCV*	0.321^∗^	0.015^∗^
*SOD* ^∗^ *MCH*	0.330^∗^	0.013^∗^
*SOD* ^∗^ *MCHC*	0.054	0.684

SOD: superoxide dismutase; WBC: total white blood cell; NEU: neutrophil; LYM: lymphocyte; MON: monocyte; EOS: eosinophil; BAS: basophil; PLT: platelet; MPV: mean platelet volume; PDW: platelet distribution width; PCT: plateletcrit; RBC: red blood cells; HGB: haemoglobin; HCT: haematocrit; MCV: mean cell volume; MCH: mean cell haemoglobin; MCHC: mean cell haemoglobin concentration. ^∗^Association is statistically significant at *p* < 0.05.

**Table 5:** Receiver operator characteristics results for predicting stroke.

Variable	Sensitivity	Specificity	Accuracy	AUC	*p* value
*Smaller test result indicates more positive test*					
SOD	0.8667	0.9333	0.9000	0.9267	<0.001
LYM	0.7333	0.9000	0.8167	0.7744	<0.001
MCHC	0.9000	0.5667	0.7333	0.7083	0.002
MCH	0.3667	0.8667	0.6167	0.6033	0.158
PDW	0.7667	0.4333	0.6000	0.5939	0.202
MON	0.8000	0.5333	0.6667	0.5711	0.338
MPV	0.5667	0.5667	0.5667	0.5361	0.630

*Larger test result indicates more positive test*					
PCT	1.0000	1.0000	1.0000	1.0000	<0.001
BAS	0.9000	0.7667	0.8333	0.8917	<0.001
RBC	0.8000	0.8333	0.8167	0.8978	<0.001
HCT	0.8333	0.7000	0.7667	0.8356	<0.001
HGB	0.7333	0.8667	0.8000	0.8133	<0.001
EOS	0.6333	0.9000	0.7667	0.7694	<0.001
NEUT	0.9000	0.5667	0.7333	0.7922	<0.001
WBC	0.5667	0.8333	0.7000	0.7294	<0.001
PLT	0.5000	0.9000	0.7000	0.7056	0.002
MCV	0.6000	0.7333	0.6667	0.6289	0.074

SOD: superoxide dismutase enzyme activity; LYM: lymphocyte; MCHC: mean cell haemoglobin concentration; MCH: mean cell haemoglobin; PDW: platelet distribution width; MON: monocyte; MPV: mean platelet volume; PCT: plateletcrit; BAS: basophil; RBC: red blood cells; HCT: haematocrit; HGB: haemoglobin; EOS: eosinophil; NEU: neutrophil; WBC: total white blood cell; PLT: platelet; MCV: mean cell volume.

## Data Availability

All data generated or analyzed during the study are included in this article.
